# Reproductive Health: Pesticides and Anencephaly

**DOI:** 10.1289/ehp.115-a78a

**Published:** 2007-02

**Authors:** Graeme Stemp-Morlock

Anencephaly is a nightmarish neural tube defect in which the fetus does not develop a forebrain, and the rest of the brain is not covered by skin or bone. Most anencephalic children die in the womb or within hours of birth. A study published in the October 2006 issue of *Occupational and Environmental Medicine* now confirms a suspected epidemiological link between parents’ occupation and this defect.

The study took place in Mexico between 2000 and 2001. Mexico has the highest occurrence of anencephaly in the world, with 8.05 cases per 10,000 live births in 2002, according to the International Clearinghouse for Birth Defects Monitoring System.

A review of fetal and infant death certificates identified cases of anencephaly of at least 20 weeks’ gestation. Of these, 157 cases were paired with 151 malformation-free cases. The mothers and fathers of the children answered questions on their age, occupation, reproductive history, food and vitamin intake, cooking methods, geographic location, and on-the-job exposure to pesticides. Principal investigator Marina Lacasaña, a professor at the University of Granada Andalusian School of Public Health, and colleagues at the Mexican National Institute of Public Health divided parental exposures into the acute risk period (from three months before the mother’s last menstruation before pregnancy through one month after) and the nonacute risk period (the time before the acute risk period).

The results showed a nearly fivefold increase in risk of anencephaly for mothers exposed to pesticides while working in agriculture during the acute risk period. Fathers who were exposed to pesticides at any time while working in agriculture had twice the risk of having an anencephalic child. Some of the more heavily used pesticides reported by the parents, including chlorpyrifos and methyl parathion, have been previously linked with possible reproductive effects.

Rudy Rull, a research scientist at the Northern California Cancer Center who has researched birth defects and pesticides exposure, believes the study has some strong points. “One of the strengths of the study is that in the exposure section they focus in on timing, which is a really sensitive issue for neural tube defects . . . especially the months before and after conception.”

However, the challenge for this and other studies will be to identify what pesticides are causing the defect. Many studies have shown that working in agriculture increases the risk of neural tube defects, says Rull, “but there’s a lot you can be exposed to [in any given farm field]. We need to know which pesticides increase the risk.”

The authors also noted there were few cases where workers wore adequate protective clothing. They hope their research will have policy implications; as they concluded in their paper, “Women involved in agricultural work, or who are living with men who work in agriculture, should be protected from direct and indirect pesticide exposure, especially during the peri-conceptional period, if they are planning to have a child.”

